# Dutch GPs’ views on prescribing mifepristone and misoprostol: a mixed-methods study

**DOI:** 10.3399/BJGP.2021.0704

**Published:** 2022-07-26

**Authors:** Julia EAP Schellekens, Claire SE Houtvast, Peter Leusink, Gunilla Kleiverda, Rebecca Gomperts

**Affiliations:** University of Amsterdam, Amsterdam.; University of Groningen, University Medical Center Groningen, Groningen.; Utrecht.; Women on Waves, Amsterdam.; Women on Waves and Women on Web, Amsterdam.

**Keywords:** abortion, induced, abortion, missed, general practitioners, mifepristone, misoprostol, Netherlands

## Abstract

**Background:**

The World Health Organization has indicated that GPs can safely and effectively provide mifepristone and misoprostol for medical termination of pregnancy (TOP). Dutch GPs are allowed to treat miscarriages with mifepristone and misoprostol, but few do so. Current Dutch abortion law prohibits GPs from prescribing these medications for medical TOP. Medical TOP is limited to the specialised settings of abortion clinics and hospitals. Recently, the House of Representatives debated shifting abortion to the domain of primary care, following the example of France and the Republic of Ireland. This would improve access to sexual and reproductive health care, and increase choices for women. Nevertheless, little is known about GPs’ willingness to provide medical TOP and miscarriage management.

**Aim:**

To gain insight into Dutch GPs’ willingness to prescribe mifepristone and misoprostol for medical TOP and miscarriages, as well as the anticipated barriers.

**Design and setting:**

Mixed-methods study among Dutch GPs.

**Method:**

A questionnaire provided quantitative data that were analysed using descriptive methods. Thematic analyses were performed on qualitative data collected through in-depth interviews.

**Results:**

The questionnaire was sent to 575 GPs; the response rate was 22.1% (*n* = 127). Of the responders, 84.3% (*n* = 107) were willing to prescribe mifepristone and misoprostol, with 58.3% (*n* = 74) willing to provide this medication for both medical TOP and miscarriage management. A total of 57.5% (*n* = 73) of participants indicated a need for training. The main barriers influencing participants’ willingness to provide medical TOP and miscarriage management were lack of experience, lack of knowledge, time constraints, and a restrictive abortion law.

**Conclusion:**

Over 80.0% of responders were willing to prescribe mifepristone and misoprostol for medical TOP or miscarriages. Training, (online) education, and a revision of the abortion law are recommended.

## INTRODUCTION

Sexual and reproductive health is a basic human right that should be acknowledged by all. Every woman has the right to choose the number, timing, and spacing of her children in a free and responsible manner, without any discrimination, violence, or coercion.^1^ To realise these rights, access to legal, safe, and comprehensive abortion care is essential.^1^^,^^2^ GPs are at the centre of the Dutch healthcare system and function as gatekeepers to specialist care. Their responsibilities include contraceptive care and other aspects of reproductive health. Historically, pregnancy-related care fell within the scope of general practice, but has since shifted to specialised care. GPs can prescribe mifepristone and misoprostol for miscarriage management; however, few actually do so as the guidelines on miscarriage do not advise it. Nevertheless, GPs are not permitted to prescribe these same medications for medical termination of pregnancy (TOP).^3^ Under Dutch abortion law, medical TOP can only be provided in special clinics or hospitals. There are 15 abortion clinics in the Netherlands, all of which are located in urban areas. Two provinces do not have an abortion clinic at all.^4^ Previous studies have recognised geographic barriers among the difficulties women face when accessing abortion care in the Netherlands.^5^^–^^7^

Left-wing Dutch political parties have proposed an amendment to the abortion law to allow GPs to prescribe mifepristone and misoprostol for medical TOP and thereby increase accessibility of abortion care. This amendment was discussed in the first months of 2022 and has been approved by the Dutch Parliament.

The World Health Organization’s safe abortion guidance indicates that GPs have the ability to effectively and safely provide mifepristone and misoprostol for medical TOP up to 9 weeks and for miscarriage management.^8^^,^^9^ The same guidance is indicated in Dutch miscarriage management guidelines.^10^ GPs already prescribe mifepristone and misoprostol for medical TOP in several other countries, including France and Ireland, with positive results.^11^^–^^13^ In these countries, medical TOP has been shown to be safe and effective, and both GPs and patients report high levels of satisfaction.^14^ Women report more control, anonymity, and privacy, and it is less expensive.^13^^,^^15^ The same was found in several states of the US, where primary care physicians were allowed to prescribe medical TOP after online consultation because of the COVID-19 pandemic.^16^

Each year, about 30 000 women in the Netherlands terminate their pregnancy.^17^ The TOP rate is 9.1 per 1000 women living in the Netherlands aged 15–49 years, which is low compared with TOP rates worldwide and with countries with similar healthcare systems. This is assumed to be the result of comprehensive sexual health education and access to contraceptives, often provided by GPs.^18^ When contraceptives fail, GPs are regularly the first point of contact for women facing an unwanted pregnancy in the Netherlands: two-thirds of women visiting an abortion clinic in the Netherlands are referred by their GP.^17^ It is hypothesised that many women would rather visit their GP when seeking medical TOP or miscarriage management than go to an abortion clinic or hospital.^19^

**Table:** How this fits in

Medical termination of pregnancy (TOP) in the Netherlands can only be provided in abortion clinics and hospitals. GPs are allowed to provide medical miscarriage management, but only a few do so. To improve access to woman-centred care, it is important to allow GPs by law to provide medical TOP. To the authors’ knowledge, this study is the first to assess Dutch GPs’ willingness to prescribe mifepristone and misoprostol for medical TOP and miscarriage management, and aims to understand anticipated enablers and barriers. These findings can help determine whether a shift in care is feasible. The findings highlight the need to revise laws and to provide training and education of medical TOP and miscarriage management.

Women benefit from access to medical TOP and miscarriage management through their GPs as it increases physical and mental autonomy.^20^^,^^21^ A shift to primary care would eliminate many existing barriers to seeking abortion care. The willingness of GPs to prescribe mifepristone and misoprostol for both medical TOP and miscarriages is vital to the success of this transition. Data on the overall willingness to provide this treatment and the barriers perceived by GPs in the Netherlands are lacking. This study aimed to gain more insight into the willingness of GPs in the Netherlands to provide mifepristone and misoprostol for medical TOP and miscarriages.

## METHOD

### Terminology

In this study the authors chose to use the terms ‘woman’ and ‘women’, to ensure legibility. Using these terms, the authors do not mean to exclude people who can become pregnant but do not identify or feel comfortable with the word ‘woman’ nor women who have been unable to conceive.

### Sampling

A systematic sampling strategy was used. Ideally, a GP from each municipality in the Netherlands would have completed the questionnaire to create a diverse and representative sample. However, a list of all GPs in the Netherlands and their contact information is not available because of privacy concerns, and it was not financially feasible to include a third party for data collection because of the non-profit status of the organisation commissioning this research. Instead, at least one GP from each municipality, chosen randomly from the Netherlands Chamber of Commerce KVK using zip codes, was approached; their data was collected online. In total, 575 invitations were sent by email to GPs, including a participation request. The questionnaire was sent via email, followed by a reminder sent 2 weeks later. The authors anticipated that not all GPs would fill in the questionnaire, and therefore that some municipalities would not be represented in the results.

Interview participants were included based on their willingness to participate. All GPs who left their contact details after finishing the questionnaire received a link to schedule a time slot for this interview; reminders were sent.

### Instruments

A mixed-methods study design was chosen to enable a holistic data capture through complementary quantitative and qualitative methods of data collection and analysis. For the quantitative part of the study, a 21-item multiple-choice questionnaire (Supplementary Appendix S1) was used to gain insight into demographics, current practices, and theoretical factors influencing willingness to provide medical TOP and miscarriage management.

The questionnaire was based on a theoretical framework. This framework, the Capability, Opportunity, Motivation and Behavioural (COM-B) model by Michie *et al*, was used to assess willingness.^22^ To validate the survey, the questions were forwarded to a pilot group and adapted based on their feedback. After completing the questionnaire, all GPs were asked if they wanted to participate in a follow-up in-depth interview. If willing to participate they could leave their contact details. The interviews were conducted until data saturation was reached.

For the qualitative part of the research, semi-structured interviews, all by phone, were conducted that allowed for in-depth topic discussion. Two authors each conducted five interviews. At the time of the interviews, one interviewer was studying international public health and the other medicine, and both had a personal interest in abortion care and strong pro-choice sentiment. The interviews were semi-structured using an interview guide with questions related to personal experience with medical TOP and miscarriage management, and a possible shift of care. A pilot interview was conducted to assess the quality of the interview guide and small amendments to the wording were made. Interviews were recorded and transcribed. Thematic analysis was carried out by each researcher, and the coding framework was validated for bias by all authors. Thematic analysis was performed to describe data in detail and identify patterns and emerging key themes.

### Statistical analysis

Data screening was executed on a univariable level to exclude missing values and outliers. Internal consistency was measured. After data screening, all results were analysed using descriptive statistics. All statistical analyses were conducted with IBM SPSS Statistics (version 27.0).

### Thematic analysis

Thematic analysis was performed on the qualitative data. All interviews were transcribed verbatim and coded in Atlas. ti (version 19). The six steps of thematic analysis, as identified by Braun and Clarke, were used.^23^ The COM-B model was used as a thematic framework to classify the barriers and enablers that were mentioned by the GPs into three themes: capability, opportunity, and motivation. All codes were clustered into these three themes by two researchers, and a third researcher was consulted for reassessment in case of disagreement. Barriers were defined as anything negatively influencing GPs’ willingness to provide mifepristone and misoprostol for both indications. Enablers were defined as anything positively influencing GPs’ willingness to provide medical TOP and miscarriage management.

## RESULTS

The first part of this mixed-methods study was a questionnaire. The overall response rate to the questionnaire was 22.1% (*n* = 127/575) and 70.9% (*n* = 90/127) of responders were women. There were 63 (49.6%) responders who had been working as a GP for >15 years. The majority of responders worked in a general practice located in Utrecht or Noord-Holland. The characteristics of all questionnaire responders are listed in Table 1.

**Table 1.** Characteristics of GPs who completed the questionnaire (*n* = 127)

**Characteristic**	***n* (%)**
**Gender**	
Woman	90 (70.9)
Man	37 (29.1)

**GP status**	
Practice owner	99 (78.0)
Paid employment	11 (8.7)
Substitute in charge^a^	16 (12.6)

**Working experience, years**	
0–5	16 (12.6)
6–10	29 (22.8)
11–15	19 (15.0)
>15	63 (49.6)

**Organisational structure**	
Healthcare centre	21 (16.5)
Multiperson practice	34 (26.8)
Duo practice	38 (29.9)
Soloist	25 (19.7)
Other	9 (7.1)

**Number of patients^b^**	
1500–1800	12 (9.4)
1801–2100	10 (7.9)
2101–2400	18 (14.2)
>2400	86 (67.7)

**Location of practice^c^**	
Groningen	3 (2.4)
Friesland	4 (3.1)
Drenthe	6 (4.7)
Flevoland	2 (1.6)
Overijssel	9 (7.1)
Gelderland	14 (11.0)
Noord-Holland	19 (15.0)
Zuid-Holland	12 (9.4)
Utrecht	21 (16.5)
Noord-Brabant	10 (7.9)
Zeeland	3 (2.4)
Limburg	2 (1.6)

a *GP who sees patients whose regular GP is absent.*

b *Missing information for 1 general practice (0.8%).*

c *Missing information for 22 general practices (17.3%).*

The qualitative part of this mixed-methods study comprised an in-depth follow-up interview. Data saturation was reached after eight interviews. Ten GPs, seven women and three men, from various Dutch provinces participated in these interviews; their characteristics are described in Box 1. Table 2 describes the outcome of the questions concerning possible barriers and enablers.

**Box 1.** Characteristics of GPs interviewed (*n* = 10)

**Code of participant**	**Gender**	**Province**	**Organisational structure practice**
R1	Woman	Zuid-Holland	Multiperson practice
R2	Woman	Noord-Holland	Healthcare centre
R3	Woman	Friesland	Soloist
R4	Woman	Utrecht	Multiperson practice
R5	Man	Noord-Brabant	Soloist
R6	Woman	Noord-Holland	Duo practice
R7	Man	Gelderland	Duo practice
R8	Man	Utrecht	Healthcare centre
R9	Woman	Noord-Holland	Soloist
R10	Woman	Utrecht	Duo practice

**Table 2.** Outcome of the questionnaire (*n* = 127)

**Characteristic**	***n* (%)**
**Miscarriage assistance request, instances**	
0–2	70 (55.1)
3–5	45 (35.4)
6–10	8 (6.3)
>10	1 (0.8)
Not sure	3 (2.4)

**Action method in case of a miscarriage**	
Self-guidance and referral	48 (37.8)
Preference to guide and treat self	31 (24.4)
Direct referral to gynaecologist	48 (37.8)

**Number of unwanted pregnancies annually**	
0–2	59 (46.5)
3–5	54 (42.5)
6–10	13 (10.2)
>10	0 (0)
Not sure	1 (0.8)

**Action method in case of an unwanted pregnancy^a^**	
Referral to abortion clinic, if no doubts	70 (55.1)
Referral to abortion clinic, after discussion	45 (35.4)
Against TOP, but will refer	6 (4.7)
Against TOP, will not refer	5 (3.9)

**Feeling qualified to provide medical TOP**	
Yes, for a miscarriage	6 (4.7)
Yes, for medical TOP	4 (3.1)
Yes, for both miscarriage and medical TOP	20 (15.7)
No, for neither miscarriage nor medical TOP	97 (76.4)

**Willing to provide medical TOP after training^b^**	
For miscarriage only	33 (26.0)
For miscarriage and medical TOP	74 (58.3)

**Access to ultrasound**	
Own practice	9 (7.1)
Midwifery practice	32 (25.2)
Other primary care facility	40 (31.5)
Referral to gynaecologist or abortion clinic	46 (36.2)

**Need for additional training**	
Yes, for miscarriages	20 (15.7)
Yes, for medical TOP	2 (1.6)
Yes, both for miscarriages and medical TOP	73 (57.5)
No, neither for miscarriages nor medical TOP	32 (25.2)

**Barriers to provide medical TOP^c^**	
No barriers	12 (9.4)
Extra administrative work	12 (9.4)
Lack of experience	75 (59.1)
Lack of time	35 (27.6)
Lack of knowledge	55 (43.3)
No access to ultrasound	44 (34.6)
Objections from colleagues	10 (7.9)
Lack of funding	16 (12.6)
Personal conviction	35 (27.6)
Public opinion (stigma)	2 (1.6)
Other	24 (18.9)

a *Missing information for 1 GP (0.8%).*

b *Missing information for 20 GPs (15.7%).*

c *Participants were free to select more than one barrier. TOP = termination of pregnancy.*

**Figure 1.**
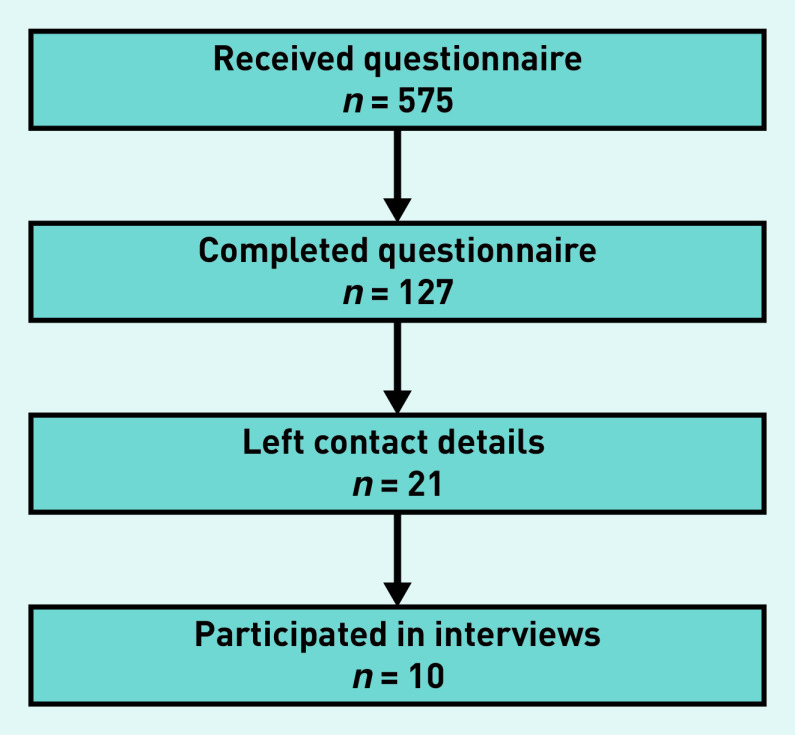
**Flowchart showing the participation rate for the quantitative and qualitative parts of the study.**

Figure 1 represents the participation rate in the different steps of this mixed-methods study.

Following the COM-B model, three themes were formulated to classify the barriers and opportunities that were mentioned by the GPs: capability, opportunity, and motivation.

### Capability

Lack of experience was mentioned by 75 (59.1%) of the GPs as a barrier to providing medical TOP and miscarriage management. Most of the responders (76.4%, *n* = 97) reported that they did not feel qualified for both. Only 15.7% (*n* = 20) already felt capable enough to prescribe the medication for both medical TOP and miscarriages.

Lack of knowledge was considered a barrier by 43.3% (*n* = 55) of responders. In total, 57.5% (*n* = 73) of responders indicated a need for training for both medical TOP and miscarriages. Only 25.2% (*n* = 32) of responders did not wish to have any training. This lack of knowledge and information was confirmed during the interviews:

*‘I think it would be useful to receive proper education with people who think alike. So everyone willing to provide medical TOP should have the possibility to participate in class and receive information about all the ins and outs. You see, most things you already know, but it never hurts to have someone with experience tell you what to expect.’*

(R5, man [M], soloist)

*‘For example, for PrEP* [pre-exposure prophylaxis] *, it is well organised. There are guidelines and a small summary chart available. That is how I would like to have it for medical TOP as well. I would prefer a plan that explains it step-by-step. I do not need to know all the details, but I want to be able to look them up.’*

(R3, woman [W], soloist)

In total, 107 (84.3%) GPs indicated that they would be willing to provide mifepristone and misoprostol after training; 26.0% (*n* = 33) would prescribe the medication for miscarriages only, and 58.3% (*n* = 74) would be willing to prescribe mifepristone and misoprostol for medical TOP.

### Opportunity

Nearly half (55.1%, *n* = 70) of responders reported seeing ≤2 miscarriages each year. Of the responders, 24.4% (*n* = 31) preferred to manage miscarriages themselves whereas 37.8% (*n* = 48) prefered to immediately refer patients to a gynaecologist. In this study, 46.5% (*n* = 59) of GPs saw ≤2 unwanted pregnancies per year, and 42.5% (*n* = 54) saw 3–5 per year. Although ultrasound dating and location of pregnancy are not required to safely prescribe mifepristone and misoprostol, 34.6% (*n* = 44) of the GPs mentioned lack of access to ultrasound as a possible barrier. Of the responders, 7.1% (*n* = 9) reported having access to ultrasound in their own practice, 25.2% (*n* = 32) had access to ultrasound diagnostics via a midwifery practice, 31.5% via other primary care facilities, and 36.2% via referral to the gynaecologist or abortion clinic:

*‘For me, one of the main barriers is the fact that I do not have ultrasound equipment in our practice. I do have midwives that work with us, but they also do not have direct access to ultrasound equipment. If this was the case, then it would be much easier. Then I would probably arrange a direct link with one of the midwives.’*

(R4, W, multiperson practice)

Under Dutch abortion law, GPs are not permitted to provide medical TOP. This was mentioned several times as an important legal barrier:

*‘I can understand that GPs are not very enthusiastic about this idea. Nobody wants to end up at the disciplinary tribunal or have to undergo a juridical procedure. You should know for sure what does and what does not belong to your field of expertise.’*

(R2, W, healthcare centre)

In order to prevent misunderstanding or violation of the law on abortion, interviewed participants stated their desire for clear guidelines on provision of medical TOP by GPs.

### Motivation

Every interview participant believed that medical TOP and miscarriage management by GPs would be beneficial for women and increase access to care:

*‘I think it can be a nice opportunity to help women in difficult situations. Because after all, the GP is a place you can always go to in case of a care request.’*

(R10, W, duo practice)

Important motivational barriers were lack of time (27.6%, *n* = 35) and lack of funding (12.6%, *n* = 16):

*‘It is not the only task that has been added to our responsibilities. There are many tasks added and none are subtracted. We are supposed to provide more care for the same number of patients and if you look at our financing structure, we receive relatively little for the extra services that we provide because we still have subscription rates.’*

(R1, W, multiperson practice)

Stigma and fear of judgement about TOP were mentioned as motivational barriers by only 1.6% (*n* = 2) of responders. However, for more than a quarter of responders (27.6%, *n* = 35), personal beliefs were a barrier to providing medical TOP.

Twelve responders (9.4%) did not anticipate any barriers to providing mifepristone and misoprostol.

## DISCUSSION

### Summary

This study aimed to explore the willingness of GPs in the Netherlands to provide mifepristone and misoprostol for medical TOP and miscarriages by obtaining more insight into enablers and barriers that influence their willingness.

This study showed that 84.3% of participating GPs were willing to prescribe mifepristone and misoprostol, and nearly two-thirds of them are willing to provide medical TOP. Lack of experience, knowledge, time, and a restrictive abortion law were the main barriers; (online) training, education, and a revision of the abortion law could address these barriers.

### Strengths and limitations

This study was able to include a diverse and heterogeneous sample in the interviews. The mixed-methods design has proven to be a strength, as the qualitative data allowed the authors to gain a deeper insight into what was highlighted in the quantitative data and consequently to further explore ideas and experiences of GPs on this topic. Although the interview sample is small (10 participants), data saturation was reached after eight interviews. The quality of the interview guide was checked by undertaking a pilot interview. All interviews were performed following an interview guide within 1 month. Thereafter, all interviews were coded and investigator triangulation was used to reduce observer bias and improve inter-rater reliability.

This study has several limitations to consider. First, because of the small sample size and low response rate, the generalisability of the quantitative results could be limited, which is a common characteristic of web-based surveys.^24^ In the Netherlands, there are 12 766 active GPs. In this sample, female GPs are slightly over-represented (80% versus 60%), compared with the overall GP population.^25^ Second, TOP can be a controversial and sensitive topic, and results should therefore be interpreted with caution because of a possible social desirability response bias. Third, responders for the interviews were sought based on their willingness to participate, causing a lack of randomisation. As none of the interviewed GPs had a negative attitude towards abortion care the interview responses do not represent the opinion of GPs who are sceptical about this topic. Lastly, all authors have a pro-choice sentiment, which could have influenced the interpretation of this research as they might be unconsciously biased when interpreting the responders’ answers.

### Comparison with existing literature

The findings of this study correspond with those of previous literature identifying a lack of accurate knowledge and skills as a barrier to providing medical TOP and miscarriage management.^26^^,^^27^ Therefore, appropriate training and online education tools for GPs to improve their affinity with the topic are necessary.^28^ Research suggests that early exposure and education about abortion care will lead to a higher acceptance rate of medical TOP provision as being part of the range of responsibilities of GPs.^29^ For this reason, training regarding the use of mifepristone and misoprostol should be incorporated into the medical curriculum.^30^

Responders identified a lack of time as one of the main motivational barriers influencing their willingness to provide medical TOP and miscarriage management. To overcome this barrier, provision of training and education tools would be beneficial.^31^ It is anticipated that because of training and exposure GPs will develop more affinity with medical TOP and realise that it does not require extra time,^32^ especially as GPs indicated they are already involved in counselling and providing aftercare to women with an unwanted pregnancy or miscarriage.

Previous studies have found that physicians’ willingness to provide medical TOP is influenced by concerns about stigma, judgement, and negative reactions,^33^ especially in conservative regions.^34^ However, in the current study, only a few participants mentioned stigma or fear of judgement as a barrier. This may be attributable to the socially progressive context in the Netherlands.

Lack of access to ultrasound diagnostics was another concern of GPs in the Netherlands. However, current standards of practice do not require ultrasound, as a positive urine pregnancy test and self-reported menstrual history have been shown to be accurate and safe means of assessing gestational age.^35^^,^^36^ Furthermore, a direct referral network to a midwifery practice or gynaecologist with access to ultrasound diagnostics already exists in the event of uncertain gestational age or suspected ectopic pregnancy.

### Implications for practice

To the authors’ knowledge, this study is the first of its kind in the Netherlands to assess GPs’ willingness to provide mifepristone and misoprostol for medical TOP and miscarriages. The findings demonstrate that the majority of responding GPs are interested in providing this care, but face barriers to doing so. In addition to revising the restrictive abortion law, policymakers must address provider-side challenges such as lack of experience, knowledge, and time.

Medical TOP and miscarriage management should become a part of the curriculum for GPs during their training and continuing medical education. The findings are particularly relevant in light of the recent debates in the Dutch House of Representatives on the amendment to allow GPs to prescribe mifepristone and misoprostol for medical TOP. During the interviews, the amendment was under consideration but had not yet been submitted for debate. The recent debates illustrate uncertainty among stakeholders and policymakers regarding opportunities for and barriers to the use of mifepristone and misoprostol in general practice. Should the amendment pass, the findings of this mixed-methods study provide implementation guidance for policymakers and can inform revision of the Dutch College of General Practitioners’ miscarriage management guidelines.

## Data

Data can be requested from the authors.
